# Clozapine as an Off‐Label Treatment for Severe and Treatment‐Resistant (Auto‐)Aggressive Behavior in a 40‐Year‐Old Patient With Mild Intellecutal Disability and Borderline Personality Disorder

**DOI:** 10.1155/crps/9991035

**Published:** 2026-01-31

**Authors:** Lukas Zabel, Nathalie Ersek, Andreas B. Hofmann, Lena Machetanz, Johannes Kirchebner, Susanne Stübner

**Affiliations:** ^1^ Department of Forensic Psychiatry, University Hospital of Psychiatry Zurich, University of Zurich, Lenggstrasse 31, Zurich, 8032, Switzerland, uzh.ch; ^2^ Department of Adult Psychiatry and Psychotherapy, University Hospital of Psychiatry Zurich, University of Zurich, Zurich, 8032, Switzerland, uzh.ch; ^3^ Department of Psychiatry and Psychotherapy, Ludwig Maximilian University of Munich, Munich, Germany, uni-muenchen.de

**Keywords:** aggressive behavior, borderline personality disorder, clozapine, mild intellectual disability, self-injurious behavior

## Abstract

**Background:**

Clozapine has shown to be effective as an off‐label treatment for aggressive behavior in patients with borderline personality disorder (BPD) and mild intellectual disability (MID). However, there is a lack of empirical evidence for its efficacy in these patient populations and results are so far heterogeneous.

**Case Presentation:**

A 40‐year‐old woman diagnosed with BPD and MID with severe and treatment‐resistant aggressive behavior was treated off‐label with clozapine. A carefully administered treatment with slow titration led to a significant reduction in self‐aggressive behavior and termination of aggressive behavior towards others over a 26‐week observation period.

**Conclusions:**

Our case highlights the potential efficacy of clozapine as an off‐label treatment for severe and treatment‐resistant aggressive behavior in patients with combined BPD and MID. Clozapine should be considered as a therapeutic option in severe and complex cases.

## 1. Background

Clozapine, a second‐generation antipsychotic agent, is best known for its efficacy in treatment‐resistant schizophrenia [1]. There is also evidence for its efficacy in the treatment of autism spectrum disorder, bipolar disorder, intellectual disability, and severe personality disorders, especially antisocial personality disorder and borderline personality disorder (BPD) [2]. Clozapine has specific effects in reducing aggressive and violent behavior, especially in patients with schizophrenia, but also in other psychiatric disorders [2]. Its anti‐aggressive effect aims mainly against impulsive‐aggressive behavior and is therefore thought to be independent of antipsychotic and sedative effects [2].

BPD and mild intellectual disability (MID) are disorders that both can result in aggressive, especially self‐injurious behavior. However, the combination of MID and BPD, which are coded as F70.1 and F60.3 according to the International statistical classification of diseases and related health problems, 10^th^ version (ICD‐10) [3], respectively, seems to be common in forensic psychiatric populations and poses considerable challenges for treatment concepts and settings. Aggressive behavior, including nonsuicidal self‐injury in these patients regularly does not respond sufficiently to various nonpharmacological and pharmacological interventions [4]. Even for the otherwise well‐proven anti‐aggressive effect of clozapine [2], there is still a lack of empirical evidence for its efficacy in these patient populations. Besides the CALMED study [5], which had to be closed early, there is a complete lack of randomized controlled trials (RCTs). Therefore, the available evidence is based solely on nonrandomized studies, case reports, and case series. Han et al. [6] for instance found limited evidence for the efficacy of clozapine in the treatment of very severe treatment‐resistant cases of BPD irrespective of comorbid intellectual disability. Similarly, Masdrakis and Baldwin [4] reported limited evidence for the efficacy of clozapine in treating severe and treatment‐resistant self‐injurious behavior in patients with BPD. A recently published review showed that 85.7% of patients diagnosed with intellectual disability and/or autism‐spectrum disorder who exhibit aggressive and self‐injurious behavior benefit from clozapine treatment [7]. Case series have also observed a significant reduction in self‐injurious behavior in patients with a comorbidity of MID and BPD following initiation of clozapine treatment [8, 9]. Real‐world data from more than 25,000 patients diagnosed with BPD in Denmark showed a significant reduction in psychiatric admissions, bed‐days, and self‐injurious behavior after clozapine initiation [10]. Similarly, real‐world data from over 26,000 Danish patients diagnosed with any form of intellectual disability (17.7% of whom had MID) showed a significant reduction in psychiatric admissions and bed‐days after clozapine initiation [11]. A reduction in self‐injurious behavior could not be demonstrated. However, this outcome was predominantly investigated in patients with a comorbid diagnosis of schizophrenia, bipolar disorder, or other psychotic disorders. There were no patients with a comorbidity of BPD and only a few patients without any psychiatric comorbidity exhibiting self‐injurious behavior (*n* = 4).

We present the case of a 40‐year‐old female forensic psychiatric patient, diagnosed with both MID and comorbid BPD, who was treated successfully with clozapine for severe and treatment‐resistant aggressive, especially self‐injurious behavior.

## 2. Case Presentation

### 2.1. Patient Description

A 40‐year‐old woman was admitted to our institution from a women’s correctional facility, to which she had been referred after being discharged from a specialized nursing home a few weeks earlier. Diagnosed with MID (ICD‐10: F70.1) and BPD (ICD‐10: F60.31), she had been under court‐mandated therapy (acc. to Art. 59 of the Swiss Criminal Code) since her conviction for multiple counts of verbal threats and physical assault 4 years ago. At the time of her admission, she had a more than two‐decade history of severe emotional instability, impulsivity, and aggressive behavior toward herself and others.

In the sense of Nock [12], the patient’s presentation of self‐injurious behavior could be most accurately characterized as nonsuicidal self‐injury accompanied by suicidal threats or gestures. The patient had no history of serious suicide attempts and she consistently reported that her self‐injurious behavior was not motivated by a genuine intention to end her life, but as a means to regulate overwhelming emotional tension.

A detailed behavioral analysis during the forensic placement showed that her (auto‐)aggressive behavior followed different patterns, thereby serving intra‐ and interpersonal functions: when experiencing intense and overwhelming negative emotions, self‐injury was a means for her to achieve short‐term relief. Furthermore, she engaged in self‐injurious behavior when the attention she received from caregivers did not seem to meet her subjective needs sufficiently. Her inpatient aggressive behavior towards others typically consisted of verbal threats and physical assaults, a pattern that as mentioned above had already led to her conviction. Another pattern was physically assaulting (often choking) caregivers with whom she fell in love. These attachments were predominantly directed toward female staff members, for whom she experienced brief and easily triggered emotional attraction. In rare instances, male caregivers were also affected. Her affective bonds were typically intense but unstable, and frequently shifted from one individual to another.

## 3. Psychiatric and Criminal Case History

The patient first exhibited behavioral difficulties at the age of 4 years while attending kindergarten. This included pronounced hyperactivity, difficulties with concentration, and an inability to complete structured tasks. These behavioral issues persisted in her school years, during which significant cognitive limitations became even more apparent. As a result, she had to repeat two grades during primary school. At the age of 14 years, the patient experienced her first psychiatric hospitalization following a severe emotional crisis. This marked the beginning of a pattern of emotional instability that would continue throughout her life.

At the age of 15 years, the patient underwent a neuropsychological assessment which resulted in an intelligent quotient (IQ) score below 70. This assessment led to a formal diagnosis of MID (ICD‐10: F70.1). Independent of this diagnosis, notable psychosocial stressors were present within her familial environment. Her sense of self‐worth was diminished due to violent episodes involving her father and feelings of neglect following the birth of a younger sibling. These adverse environmental factors likely contributed to the development of a maladaptive personality structure characterized by an intense need for attention and external validation.

Initially, the patient’s self‐destructive behavior, distractibility, lack of concentration, restlessness, and excessive activity were subsumed under a diagnosis of early childhood hyperkinetic conduct disorder. At the age of 16 years, this diagnosis was refined to attention deficit hyperactivity disorder (ADHD) (ICD‐10: F90.0), reflecting the persistence of her symptoms and their impact on her functioning.

By adolescence, the patient began exhibiting emotional dysregulation, impulsivity and self‐injurious behavior. BPD (ICD‐10: F60.31) was first diagnosed at the age of 22 years. The patient showed marked mood shifts with recurrent episodes of intense anger disproportionate to situational triggers, typically arising in the context of frustration or perceived rejection. When interpersonal stress occurred, including conflicts with nurses or staff, the patient would intermittently escape from institutions and occasionally consume heavy amounts of alcohol. Her interpersonal relationships were characterized by a high degree of instability, typically alternating between idealization and devaluing others, and were accompanied by efforts to prevent perceived abandonment. Her self‐concept was fragile and characterized by a chronic sense of emptiness and low self‐esteem. Overall, the patient clearly met the diagnostic criteria for BPD (ICD‐10: F60.31) exhibiting the following symptoms: marked impulsivity with a tendency to act without considering the consequences; marked tendency to interpersonal conflicts while being involved in intense and unstable relationships; excessive efforts to avoid abandonment; a liability to outbursts of anger and aggressive behavior with an inability of behavioral control; recurrent threats and acts of self‐injurious behavior; unstable mood with frequent shifts; disturbances in self‐image and identity; chronic feelings of emptiness. Since reaching adulthood, the patient had been hospitalized almost continuously due to the severity of her symptoms, including emotional instability and self‐aggressive behavior. These hospitalizations often followed interpersonal conflicts or perceived rejection, due to the central role of relational triggers in her symptomatology.

At the age of 22 years, these difficulties culminated in her first criminal conviction. Since then numerous further convictions followed, mostly for threats and physical assaults. These incidents often arose from intense interpersonal conflicts, marked by a recurring pattern of forming strong but ephemeral emotional attachments to female caregivers and reacting aggressively when these attachments were not reciprocated in the way subjectively expected by the patient. Her first recorded offense occurred during hospitalization where she assaulted a psychologist and threatened staff. That year, she also attacked a prison officer, which led to court‐mandated therapy 1 year later. Despite extended therapy, her behavior, including threats and physical assaults, persisted. Her criminal history reflects challenges central to her condition, particularly emotional dysregulation and interpersonal conflicts linked to her BPD. At the age of 31 years, she was conditionally released to a care facility, but frequent psychiatric crises and aggressive incidents continued.

Notable was an incident 3 years later in which she threatened a caregiver with a knife, following a conversation the day before in which the caregiver informed her that her medication with methylphenidate needs to be discontinued. The patient reacted with heightened tension and subsequently threatened the victim with a knife. Subsequent years saw repeated threats and assaults, leading to a 12‐month prison sentence and a new therapy mandate at the age of 35 years. 4 years later, ongoing self‐injurious behavior, especially ingesting inedible objects necessitated her transfer to the high security ward specialized in forensic psychiatric patients for further treatment under intensive control.

Over the years, the patient’s treatment history included a wide range of pharmacological interventions aimed at managing her emotional instability, impulsivity, and aggressive behavior. She had been prescribed various antipsychotics, including risperidone, quetiapine, and olanzapine, as well as benzodiazepines such as diazepam, lorazepam, and alprazolam. Additionally, mood stabilizers, like valproate and lamotrigine, had been trialed. Despite these interventions, the patient had so far experienced either no improvement or only limited efficacy in controlling her symptoms, with aggressive behavior remaining particularly resistant to pharmacological management.

At the age of 30 years, the patient was first treated with clozapine at dosages of up to 300 mg/day over a period of 20 months. Clozapine was then discontinued due to unpleasant side effects, especially nocturnal enuresis and urinary incontinence. However, several reports from this period describe its anti‐impulsive and anti‐aggressive effect. The patient herself described good memories of clozapine’s calming and anti‐aggressive effect but was afraid of the unpleasant side effects.

### 3.1. Examination Results

The patient’s height was 150 cm and her weight 73 kg leading to a body mass index (BMI) of 32.4 kg/m^2^. She smoked 20 cigarets a day. A physical examination revealed several abnormalities: she showed signs of facial dysmorphia with a very slim upper lip (Lip philtrum guide grade 5) and a flattened philtrum (Lip philtrum guide grade 4). The length of her palpebral fissure was 2.8 cm, which is in the normal range (>3^rd^ percentile). Furthermore, she showed a postural‐ and action tremor of high frequency and low amplitude, a very mild left‐sided dysdiadochokinesia and a rigidity of the upper extremity. Laboratory tests showed no abnormalities despite hypochromic, microcytic erythrocytes due to iron deficiency. An electroencephalogram (EEG) showed no abnormalities. Magnetic resonance imaging (MRI) of the brain revealed a generalized atrophy, particularly of the frontoparietal and temporo‐hippocampal areas. Genetic testing detected a variant of uncertain significance (c.64 G>A p. Val22Ile) in the *RABA1* gene (heterozygote) which plays a critical role in neuronal morphogenesis and has been associated with neurocognitive disorders [13]. The patient had taken IQ tests twice at the age of 15 and 29 years and scored below 70 both times, leading to the diagnosis of MID according to ICD‐10 (F70.1). Due to the normal length of the patient’s palpebral fissure, she had only two of the three facial features needed for a diagnosis of fetal alcohol syndrome (FAS). Due to the lack of information on the mother’s alcohol consumption during pregnancy, a partial FAS (pFAS) could not be ruled out. Overall, the etiology of the patient’s MID remained unclear.

In summary, an underlying organic brain disorder seemed to be probable, but could not be proven with certainty. Because the brain damage was believed to have occurred before or during birth, a personality disorder due to brain damage (ICD‐10: F07) could be ruled out, since there was no premorbid personality that could have changed. The clinical picture was dominated by both an intellectual disability with behavioral abnormalities, as well as emotional instability, impulsivity, aggressive, and autoaggressive behavior. The patient’s behavioral abnormalities clearly followed the borderline pattern, thereby justifying and confirming the previous diagnoses of MID (ICD‐10: F70.1) and BPD (ICD‐10: F60.3).

### 3.2. Course of Treatment at the Center of Forensic Inpatient Therapy

The patient was admitted to our high‐security ward at the age of 40 years. In the 34 weeks between admission and start of treatment with clozapine (period 1, before clozapine treatment, shown in Table 1), there were 30 incidents of self‐aggressive behavior and seven incidents of aggressive behavior towards others, amounting to a rate of 0.88 incidents of self‐aggressive behavior per week and one incident of aggressive behavior every 5 weeks. Of these 30 incidents of self‐aggressive behavior, 24 were incidents of ingesting inedible objects, for example plastic bottle caps, batteries or pieces of clothing and six were incidents of head banging against a wall or door. Of the seven incidents of aggressive behavior towards others, four were physical assaults against caregivers or fellow patients and three were verbal death threats. In the last 3 months before starting clozapine, the rate of incidents of aggressive and self‐injurious behavior escalated: 19 of the 30 incidents of self‐aggressive behavior, that is, 1.5 incidents per week occurred during these months. Eight weeks prior to starting clozapine, there was a major incident in which the patient physically attacked a caregiver, pulled him to the floor and broke his midfoot. This increase in aggressive behavior was explained by heightened stress levels during this period. The expected end of court‐mandated therapy created significant uncertainty about the patient’s future care, which she found highly distressing. This triggered fears of abandonment and loss of structure, further destabilizing her emotionally and contributing to maladaptive and aggressive behavior.

**Table 1:** Incidents of aggressive behavior before and during clozapine treatment in different treatment settings.

Incidents of aggressive behavior	Period 1: before clozapine treatment (high security ward, 34 weeks)	Period 2a: under clozapine treatment (high security ward, 15 weeks)	Period 2b: under clozapine treatment (nursing home, 11 weeks)	Period 2: period 2a + 2b (26 weeks)
Self‐aggressive behavior (total number of incidents)	30	0	3	3
Self‐aggressive behavior (incidents per week)	0.88	0	0.27	0.11
Aggressive behavior towards others (total number of incidents)	7	0	0	0
Aggressive behavior towards others (incidents per week)	0.2	0	0	0

During her stay in the high security ward, several medications were tried in order to improve her emotional regulation and reduce her aggressive behavior. However, all these medications, including antipsychotics (paliperidone up to 3 mg/day, quetiapine up to 100 mg/day, pipamperone up to 80 mg/day, zuclopenthixol up to 50 mg/day, and levomepromazine up to 150 mg/day), antidepressants (escitalopram up to 10 mg/day), and benzodiazepines (diazepam up to 30 mg/day and alprazolam up to 1 mg/day), did not show sufficient effects, although the patient had been treated with up to six psychopharmacological drugs simultaneously.

Ultimately, due to the treatment‐resistance of her symptoms and a constantly high rate of aggressive, especially self‐injurious behavior the patient was offered an off‐label treatment with clozapine. The patient ultimately consented. To avoid side effects at best, clozapine was titrated slowly with a rate of 25 mg/day per week (12.5 mg/day twice per week). Following Han et al. [6], we chose a target dose of 200 mg/day which was reached after 8 weeks. At a daily dose of 200 mg/day, plasma levels lay between 210 and 230 ng/mL, which is one‐third to one‐half of the plasma levels typically targeted in patients with schizophrenia. Side effects that the patient experienced were lighter forms of tachycardia, hypersalivation, and obstipation, all of which were well controlled by symptom‐orientated management, that is, bisoprolol, ivabradine, atropine drops, and macrogol. She did not experience nocturnal enuresis again, what she had previously suffered from during the first therapy attempt with clozapine. During the titration period, two more incidents of aggressive and autoaggressive behavior occurred: on Day 3, while on a clozapine dose of 12.5 mg/day, the patient swallowed cigaret butts and domino stones and uttered death threats against caregivers. On Day 38, while on a clozapine dose of 137.5 mg/day, she swallowed a pen. After reaching a daily clozapine dose of 150 mg/day, the patient showed no more aggressive or self‐injurious behavior for the next 15 weeks until her discharge (period 2a, under clozapine treatment, high‐security ward, shown in Table 1). This temporary termination in aggressive behavior was mediated by a marked improvement in the patient’s ability to regulate her negative emotions: when experiencing stress and negative emotions she was now able to “endure” them, and therefore, gained time to think about alternative coping strategies, for example, talking to caregivers, taking medication, et cetera. The pressure to act out her emotions by self‐injury or assaulting others was lowered. She managed to not engage in aggressive behavior for 15 weeks, despite uncertainty how life will go on after release and ending of her court‐mandated therapy, and facing a lot of interpersonal and environmental stress, for example, conflicts with her mother, a caregiver with whom she once fell in love, and her lawyer. The patient herself noticed a positive effect on her emotional stability, impulsivity, and aggressiveness, and was, therefore, motivated to continue the treatment with clozapine. Her constant stability allowed for simultaneously reducing her daily diazepam dose from 20 to 10 mg. Further dose reductions were precluded by her increasing need for on‐demand medication.

In addition to pharmacological treatment, the patient benefited significantly from the therapeutic environment and interventions in the high‐security ward. She regularly participated in milieu therapy, including daily routines, regular goal‐oriented discussions with nursing staff, and occupational therapy. Individual therapy focused on a token economy, simplified emotional and behavioral analysis, and emotion regulation strategies. A personalized skills box with tension‐reducing items was created. The token system reinforced positive behavior and was linked to meaningful rewards. Therapeutic agreements helped minimize isolation, supporting trust, motivation, and emotional stability.

### 3.3. Follow‐Up

The patient was discharged to a nursing home after 12 months of inpatient treatment. She felt uncomfortable there because of the unfamiliar living environment and care team. After 1 week, she threatened to swallow objects twice, but never did. After 2 weeks, she started threatening to stop taking her medication. After another threat to swallow objects, she was finally admitted to a psychiatric hospital. She returned after 1 week and remained stable for the next 3 weeks, taking her medication and never showing any aggressive behavior. After more than 6 weeks, she swallowed a plastic object and was again admitted to a psychiatric hospital. In the meantime, the nursing home had terminated her contract. Since then, she has been admitted to different inpatient wards of a general psychiatric hospital. The dose of clozapine was increased to 250 mg/day. During the second week of her hospital stay, she twice swallowed objects (pieces of a compact disc and two small batteries). After that, her condition remained stable and she did not again engage in self‐aggressive behavior for the next 5 weeks. In summary, in the 11‐week period following her discharge (period 2b, under clozapine treatment, nursing home, shown in Table 1), there were a total of three incidents of self‐aggressive behavior (ingesting objects) and no incidents of aggressive behavior towards others.

In the 26‐week period after reaching a sufficient clozapine dose (period 2, under clozapine treatment, shown in Table 1), the patient engaged in self‐aggressive behavior a total of three times and never engaged in aggressive behavior towards others. Compared with the 34 weeks prior to clozapine treatment (period 1), this represents a 69% (period 2b), 87.5% (period 2), or even 100% (period 2a) reduction in self‐aggressive behavior and a 100% reduction in aggressive behavior towards others.

This marked reduction in aggressive behavior experienced by the patient after the introduction of clozapine is visualized in Table 1.

## 4. Discussion

Our case describes a 40‐year‐old woman diagnosed with MID and BPD who was successfully treated off‐label with clozapine for severe and treatment‐resistant (auto‐)aggressive behavior.

Aggressive behavior in patients diagnosed with BPD and/or MID poses a great therapeutic challenge. In clinical practice, these patients are often unsuccessfully treated with different drugs, as happened with the patient described here, including antipsychotics, mood stabilizers, and antidepressants resulting in polypharmacy with an underestimated risk of side effects.

Although clozapine is known for its antiaggressive effects and has been used as an off‐label treatment for severe and treatment‐resistant aggressive behavior in patients with BPD and/or MID [2, 6], the evidence is limited since it is based on case series and reports.

Unfortunately, the first RCT investigating the use of clozapine in patients with BPD (“CALMED study”) had to be closed early due to the COVID‐19 pandemic [5]. Case series have observed a significant reduction in self‐injurious behavior in mildly intellectually disabled patients with BPD following initiation of clozapine treatment [8, 9]. The case described here adds to this existing evidence.

This case highlights that aggressive behavior in patients with BPD and/or MID is always multifactorial, determined by an interplay of biological, environmental, and interpersonal factors. The patient experienced a significant improvement in her well‐being with a 15‐week period (period 2a, shown in Table 1) without aggressive behavior after the administration of clozapine (a change in a biological factor) while her treatment environment (high‐security ward) remained unchanged. Then, presumably due to these altered (neuro‐)biological conditions, she was able to cope with interpersonal stress and resulting negative emotions without engaging in any aggressive behavior. After her discharge, she faced altered environmental circumstances and started again to engage in self‐aggressive behavior, albeit to a lesser extent (period 2b, shown in Table 1). In the patient’s case, clozapine as a “biological intervention” raised the threshold for engaging in aggressive behavior. Importantly, from a forensic perspective, she never again engaged in aggressive behavior towards others after starting clozapine. As female forensic patients cause more inpatient aggression and more severe aggressive incidents than male patients [14], the expanded use of clozapine as an off‐label treatment may help to reduce the number of aggressive incidents and increase safety in forensic psychiatric settings.

Furthermore, the case highlights the significance of environmental factors in reducing aggressive incidents, especially the presence of a stable environment.

Clozapine interferes with brain networks that are involved in emotion, thinking, action planning and their interplay. It has been hypothesized that clozapine’s unique receptor binding profile and influence on serotonergic transmission mediates its anti‐aggressive effects [2]. Studies in patients with BPD have shown a connection between prefrontal serotonergic dysfunction and impulsive‐aggressive behavior [15]. Its anti‐aggressive effect is mainly mediated by its anti‐impulsive effect. Clozapine has shown efficacy in the treatment of impulsive‐aggressive behavior irrespective of the underlying psychiatric disorder.

Due to its potentially fatal side effects, many physicians are hesitant to prescribe clozapine, even as an on‐label treatment for schizophrenia, but even more as an off‐label treatment for other psychiatric conditions. However, as was done in our case, at least the risk for inflammatory adverse effects, for example, myocarditis, nephritis, or drug reaction with eosinophilia and systemic symptoms (DRESS), can be reduced by choosing a slow tapering rate [16]. Ultimately, the risks of clozapine’s side effects must be weighed against the potential benefits, keeping in mind the side effects of polypharmacy and some specific side effects of mood stabilizers. However, unlike during her first treatment, the patient tolerated clozapine well, did not develop any urological symptoms and experienced a 15‐week period without aggressive behavior and a marked increase in her overall well‐being.

This case demonstrates the potential efficacy of clozapine as an off‐label treatment for severe and treatment‐resistant aggressive behavior in patients with combined BPD and MID. A carefully administered treatment with slow titration led to a marked reduction of self‐aggressive behavior and a termination of aggressive behavior towards others within a 26‐week observation period. This clinical observation highlights the importance of considering clozapine as a therapeutic option in severe and complex cases.

## Funding

Open access publishing facilitated by Universitat Zurich, as part of the Wiley ‐ Universitat Zurich agreement via the Consortium Of Swiss Academic Libraries.

## Consent

Both the patient and her legal guardian gave their written consent that the patient‘s medical history could be used to create this case report. Additionally, personally identifiable details have been removed whenever possible.

## Conflicts of Interest

The authors declare no conflicts of interest.

## Data Availability

The data that support the findings of this study are available upon request from the corresponding author. The data are not publicly available due to privacy or ethical restrictions.
